# Proteomic Changes to the Updated Discovery of Engineered Insulin and Its Analogs: Pros and Cons

**DOI:** 10.3390/cimb44020059

**Published:** 2022-02-11

**Authors:** Naeema Hanif, Hezhou Wu, Peizhou Xu, Yun Li, Amir Bibi, Asma Zulfiqar, Muhammad Zafar Iqbal, Muhammad Tahir, Xiangyang Zhang, Asif Ali

**Affiliations:** 1 State Key Laboratory of Crop Gene Exploration and Utilization in Southwest China, Rice Research Institute, Sichuan Agricultural University, Chengdu 611130, China; naeemahanif910@gmail.com (N.H.); xpzhxj@163.com (P.X.); 2 Department of Biomedical Sciences, National University of Science and Technology, Islamabad 44000, Pakistan; 3 Hunan Taohuayuan Agricultural Technologies Co., Ltd., Yueyang 415000, China; wuhezhou@163.com; 4 Chengdu Academy of Agricultural and Forestry Sciences, Chengdu 611130, China; liyunxpzh@163.com; 5 Department of Plant Breeding and Genetics, University of Agriculture, Faisalabad 38000, Pakistan; ameerbibi@gmail.com; 6 Department of Botany, Quaid-e-Azam Campus, University of Punjab, Lahore 05422, Pakistan; asma.botany@pu.edu.pk; 7 College of Grassland Science and Technology, Sichuan Agricultural University, Chengdu 611130, China; m.zafarsindhu@hotmail.com (M.Z.I.); meemmerani@hotmail.com (M.T.); 8 Branch of China National Hybrid Rice Research and Development Centre, Sichuan Tiland Huizhi Biology Science and Technology Co., Ltd., Chengdu 611130, China

**Keywords:** insulin, insulin analogs, hypoglycemia, diabetes mellitus, hemoglobin A1C

## Abstract

The destruction of β-cells of the pancreas leads to either insulin shortage or the complete absence of insulin, which in turn causes diabetes Mellitus. For treating diabetes, many trials have been conducted since the 19th century until now. In ancient times, insulin from an animal’s extract was taken to treat human beings. However, this resulted in some serious allergic reactions. Therefore, scientists and researchers have tried their best to find alternative ways for managing diabetes with progressive advancements in biotechnology. However, a lot of research trials have been conducted, and they discovered more progressed strategies and approaches to treat type I and II diabetes with satisfaction. Still, investigators are finding more appropriate ways to treat diabetes accurately. They formulated insulin analogs that mimic the naturally produced human insulin through recombinant DNA technology and devised many methods for appropriate delivery of insulin. This review will address the following questions: What is insulin preparation? How were these devised and what are the impacts (both positive and negative) of such insulin analogs against TIDM (type-I diabetes mellitus) and TIIDM (type-II diabetes mellitus)? This review article will also demonstrate approaches for the delivery of insulin analogs into the human body and some future directions for further improvement of insulin treatment.

## 1. Introduction

Insulin is an anabolic hormone, produced by the β-cells of the pancreas, and is necessary for monitoring the metabolism of carbohydrates. It operates by increasing the transport of glucose from the bloodstream into the muscles and adipose tissues after the ingestion of food. It also maintains the rate of glucose formation required by the body during the fasting period [1]. The gene of human insulin possesses three exons and two introns and is located on the p arm of chromosome No.11, designated as 11p15.5 [2]. To express the gene of insulin protein through β-cells of the pancreas, a segment of DNA containing sequences of about 350 nucleotides situated on the above site of the starting point of transcription is needed [3]. Proinsulin (the inactive form of insulin) weakly interacts with the receptor of insulin hormone, and its biological activity is exceptionally (~5%) lesser [4]. The removal of the C peptide is critical for appropriate pleating of insulin, generating the biologically active hormone [5]. So, the removal of the C-chain occurs through the specified pro-hormone convertase enzymes (i.e., (PCI (Pro-hormone convertase I) and PC-II (Pro-hormone convertase II)), during transportation from an organelle (i.e., the Golgi apparatus toward immature secretory organelles) [4]. The C-terminal Arg, Arg amino acids of the B-chain are excised through carboxy peptidase H. After that, Zn+ binds to the newly synthesized insulin, forming hexamers within peculiarized secretory granules for preservation. Zn+ also facilitates the protection against denaturation and misfolding of insulin protein [6]. The life span of insulin is about 5–7 min in circulation. The release of insulin between 0.5 and 1.0 unit/hour is enough to sustain normal metabolic action and confine haptic glucose formation during food ingestion [7]. The feedback mechanism highly monitors the secretion of insulin hormone (e.g., avoiding conditions such as hypoglycemia, hyperglycemia, the breakdown of proteins, lipids, and the formation of ketone bodies). Big molecules of starch present in the food are digested and form glucose molecules. β-cells have receptors called glucose type-II (GLUT-2) [8] receptors on their surface. Through this receptor, β-cells sense glucose molecules, which in turn secrete insulin molecules into the bloodstream to provoke the uptake of biomolecules into the other cells. Insulin inhibits the gluconeogenesis (formation of new glucose molecules from its breakdown products) and glycogenolysis (glycogen breakdown) in the liver, thereby reducing the blood glucose level and, in turn, causing the β-cells of pancreas to halt the production and secretion of insulin. However, any shortage in insulin hormone or elevated insulin insensitivity causes an imbalance in the metabolic process, which can cause a life-threatening disorder known as “diabetes mellitus” [9,10]. The death rate in 2011 was about 8.2% just due to diabetes mellitus; in 2017, approximately 5 million deaths throughout the world of people in the age range of 20–99 years age was estimated [11,12]. This disorder occurs in two forms, namely type-I diabetes mellitus and type-II diabetes mellitus. Type-I diabetes mellitus involves the synthesis and secretion of insulin from β-cells which become deteriorated through the immune system [13]. Those having type-II diabetes Mellitus would have some defects in the β-cells for the secretion of insulin [14]. Both conditions upsurge the continuous process of gluconeogenesis and glycolysis in the liver, resulting in elevated blood and urine glucose levels. On the contrary, elevated concentrations of insulin can repress gluconeogenesis, thus enhancing peripheral penetration of glucose and cause an emergent medical condition called hypoglycemia [1]. External insulin is the only option for treating patients with type I and II diabetes, showing the importance of insulin and its analogs [15,16]. Since the discovery of insulin, insulin analogs were produced through GMOs (genetically modified organisms) after a long period that empowered better compatibility between the insulin activity profiles and fluctuations in glucose extent [17]. The word analog means “similar to”, which shows that insulin analogs are similar to human insulin but not the exact copy of human insulin. There are short, fast, and long-acting insulin analogs. Also, the mixture of these two or three to increase the effectivity of such insulin analogs. Such insulin analogs copy naturally producing human insulin. Some modifications have been carried out by changing or replacing amino acid sequences to produce some desirable features in such analogs. Previous efforts have already highlighted recent trends, structural changes, and academic insights into the engineering and delivery of insulin and its analogs [18,19,20,21,22]. However, the earlier efforts did not explain the detailed possible consequences of insulins preparation and usage. Our review entails the benefits and risks of insulin usage in daily life in addition to its analog’s development and proteomic changes.

## 2. Background of Insulin Preparations

**Figure 1:**
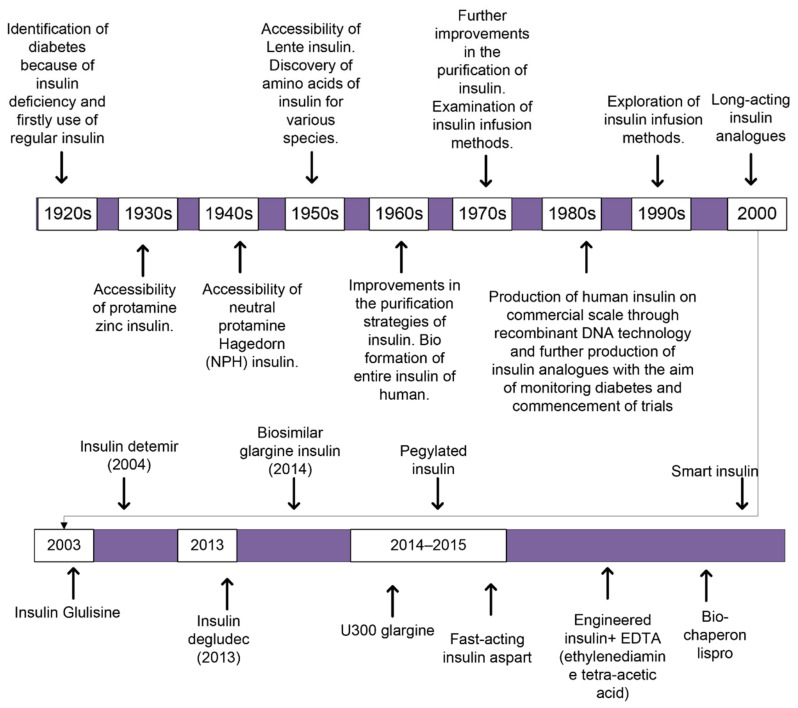
A schematic block-diagram reveals how treatment against diabetes through insulin is improved over time from 1920s up to now. With the introduction of new technologies in the field of biotechnology, how different insulin analogs were discovered and bring improvements in the remediation against diabetes either TIDM or TIIDM.

The insulin subjected to the first patient with diabetes was the unpurified extract of an animal’s pancreas. It declined the blood glucose level but mostly caused severe infections. Afterwards, many changes had been incorporated with the insulin over the century for the first use of insulin in humans, as shown in (Figure 1). Several trials were conducted to delay the resorption and to minimize the number of injections required per day. Such trials led to the production of Lente, semi-Lente, ultra-Lente, Monotard (70% Ultra Lente, 30% semi-Lente) and protamine zinc insulin (PZI) that were used for treatment purposes until 2004/2005. Neutral protamine Hagedorn (NPH) replaced the previous analogs [23]. Through the advanced technologies such as recombinant DNA technology, human insulin hormone is modified and resulted in the adapted characteristics, which involve physicochemical, biological, and pharmacodynamic properties, producing molecules called insulin analogs [24]. Designing insulin analogs has aided our capability to understand the complex processes associated with their development and use [24]. In the 1980s, patients were mostly treated with a solution of NPH or zinc-dependent insulin that was injected before breakfast and dinner daily [16]. Presently, few pharmaceutical industries are developing insulins from animals for human purposes as a result of high prices and concerns regarding well-being. Alternatively, many industries are producing biologically artificial human insulins employing recombinant DNA technology.

Within the 1960s and 1970s, the human insulin produced through modern technology (recombinant DNA technology), progressed. In 1982, it was certified for medicinal purposes. The antecedent protein to biologically artificial human protein is produced through genetically engineered organisms and proteolytically digested to form biologically active insulin. Presently, about 70% of insulin is synthesized through recombinant DNA technology and sold throughout the world. Consequently, NPH, Lente and ultra-Lente were remanufactured using human insulin. As clinicians finally preferred NPH insulin and other basal insulin preparations rather than ultra-Lente, the practice of ultra-Lente among subjects reduced over time. Its synthesis was stopped in 2005 by Eli Lilly [25].

Insulin analogs are classified based on their PK properties as quick, long, short, and intermediate. The insulin preparations having rapid and shorter action have been taken as bolus doses (quick bursting of insulin), and preparations having moderate and long action have been taken as basal doses (normal dosage).

## 3. Insulin Preparations Having a Lesser Period of Action

### Regular Insulin

**Figure 2:**
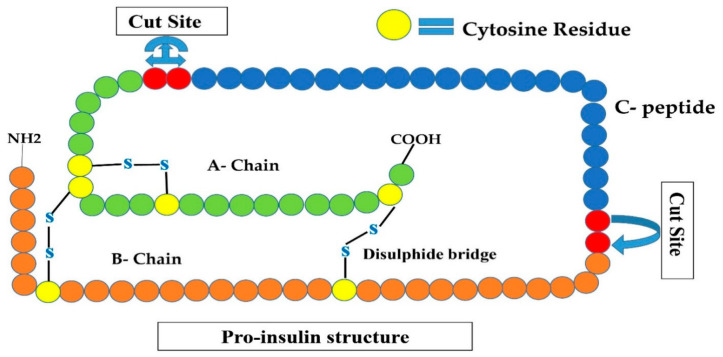
Shows the proinsulin structure that is an inactive form of insulin (A-chain, B-chain, and C-peptide). Convertases I and II remove C-peptide by cleavage at two sites are shown by red circles, converting this inactive insulin to active human insulin.

This type of insulin is a primary accessible insulin analog and hence represents the first short-acting insulin molecule. The structure of this analog is similar to the insulin manufactured by the β-cells (i.e., six units of insulin, each having A and B-chain interconnected by two disulfide linkages (an extra disulfide link between the two amino acids of A-chain), that are surrounding a zinc ion forming a hexamer (six units), as shown in (Figure 2)). When such insulin is administered intravenously into the blood circulation, these six bounded units instantly segregate into single units and can bind with the insulin receptor on interested tissue and immediately lowers blood glucose level. When it is administered into the subcutaneous tissue (i.e., under the skin surface), the hexamers must segregate into single units before the occurrence of absorption into the bloodstream, thereby delaying the beginning of the activity of glucose-reducing influence [16,26]. It happens due to different factors such as injected position, blood circulation, and thermal conditions causing alterations in operation and inconsistency among the profiles of insulin operations. Based on this mismatch, it is advised that the patient take the injection of regular insulin about 15–30 min before taking a meal [16]. Mostly, patients do not follow this instruction, considering the inconvenience and difficulties with the appointed period of meal [16,26]. The inclusion of zinc or protamine to the regular insulin resulted in the generation of zinc and NPH insulins, respectively. This addition into the insulin results in the generation of “lumps”, where insulin components are bounded to these substances, causing their action contour to be prolonged [27].

In 20 years back, insulin preparations having fast and long-lasting action have been discovered to copy the naturally produced insulin molecule through β-cells of the pancreas as opposed to that of formerly obtained [1,16]. Three swiftly operated insulin analogs are accessible for medical purposes in Europe and USA; insulin Lispro, insulin aspart, and insulin Glulisine having quick action [23].

## 4. Insulin Preparations Having Quick Action

### 4.1. Lispro Insulin

**Figure 3:**
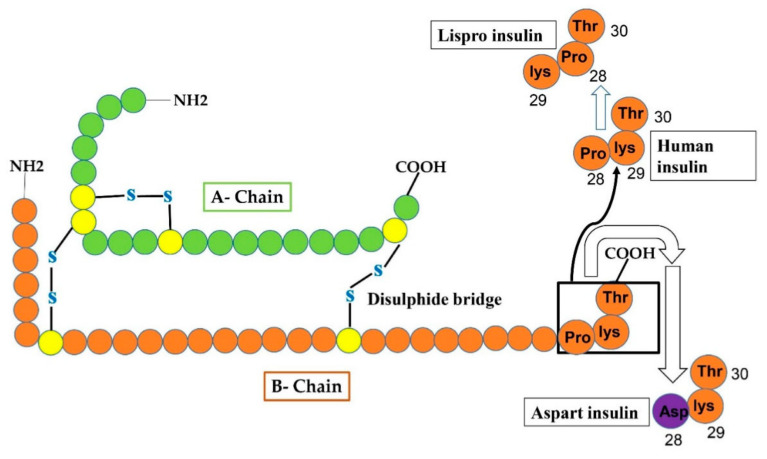
Reveals that changes in human insulin produce insulin Lispro and insulin aspart. In case of Lispro, lys and pro amino-acid residues at position 29 and 28 of the B-chain, respectively, of human insulin interchange (pro at 29 and lys at 28 site) to form insulin lispro. In aspart insulin, aspart residue (shown as purple circle) is added to site-28 of B-chain instead of proline (at 28-site) that was originally present in human insulin.

The first quick-acting insulin analog was discovered in 1996, called insulin lispro, commercially available in the market, and biologically modified in a way that the ultimate Lys and pro amino acids on the C-site of the B-chain ware inverted as shown in (Figure 3). The amino acid substitution aims to validate the durability of monomers with quick segregation and resorption after sub-cutaneous administration of insulin [28]. Such modification does not change the receptor-binding affinity yet efficiently halts the production of insulin dimers and hexamers, permitting a higher quantity of functional single unit insulin to be quickly accessible for meal regimens. Insulin lispro facilitates rapid subcutaneous resorption, prompt and better insulin peak, a brief timespan of action and greater management of glucose excursions after a meal as opposed to regular human insulin [29]. Anyhow, subjects with diabetes using insulin lispro may undergo hypoglycemic conditions if they do not take a meal for 15 min after taking medicine. Moreover, if a meal is deficient in carbohydrate contents, postprandial hypoglycemia may happen. Hence, the dosage of insulin lispro must differ based on meal constitution and extent [30].

### 4.2. Insulin Aspart

Novo Nordisk commercialized an analog of insulin having quick action as Novolog/Novo Rapid. In such analog, the residue proline at position 28 of the B-chain has been substituted with an aspart residue as shown in (Figure 3) allowing elevated charge repulsion to intercept additional hexamer production [31]. In June 2000, US Food and Drug Administration (FDA) approved the commercialization of insulin aspart [32]. The start of operation is about 15 min, the apex effect is obtained within about 45–90 min, and the period lasts for 3–5 h [33]. Since insulin aspart has a lower affinity for plasma proteins, it is released from the bloodstream faster, with an average shelf life of 81 min vs. 141 min for regular human insulin. [33]. The composition of aspart insulin is pharmaceutically identical to that of the insulin lispro having glycerin, metacresol zinc and phenol, and has disodium hydrogen phosphate acting as a buffer [34,35]. Many studies show that the influence of insulin lispro and insulin aspart is the same on minimizing blood glucose extent without any variability in the time to excessive insulin volume [34,36,37].

### 4.3. Insulin Glulisine

**Figure 4:**
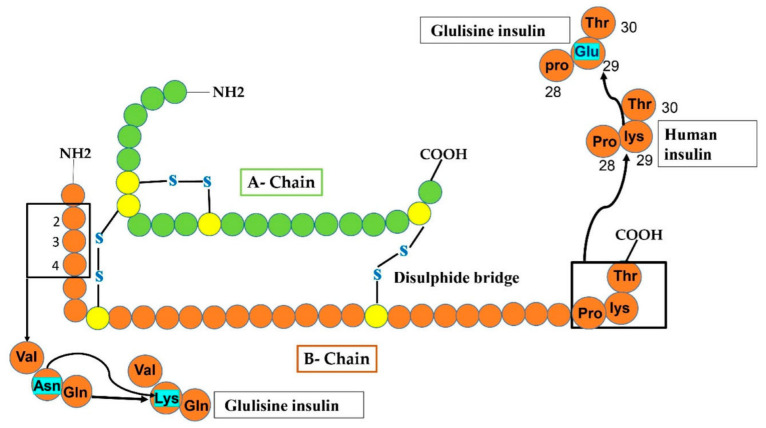
In Glulisine analog, replacement occurs at two sites of the B-chain after the modification of human insulin. At carboxy-terminal, glulisine residue substituted lys residue at position 29, while at amino-terminal, lysine amino acid substituted Asn (shown by sky-colored rectangles).

Analog of insulin produced through recombinant technology has a fast action, formulated using non-diseased in vitro strain of E-coli (K12). In insulin Glulisine, lysine residue takes the place of asparagine at position 3 of the B chain and lysine at position 29 of the B chain was superseded with glutamic acid as shown in (Figure 4) [38]. Though zinc is necessary for the maintenance of hexameric form for obtaining feasible half-life for lispro and aspart, [39] the oligomeric constituents of Glulisine are fixed without the zinc incorporation seemingly due to unchanged proline amino acid at site B28 causing molecular dimerization [40]. For subcutaneous administration of insulin Glulisine, it must not be fused with insulin analogs except NPH insulin. Mostly, it is subjected up to 15 min before the meal or within 20 min after beginning a meal. In short, rapid-acting insulin analog has resorption within 10–15 min after injection under the skin, peak action is 30–90 min, and the timespan of action is 4–6 h, imitating natural physiological prandial insulin secretion. Nonetheless, it is suggested to use fusion of insulins having quick action along with the insulin preparations having a long duration of action to improve suitable control of glycemia [33].

## 5. Insulin Preparations Having Long Term-Action

The two long-acting insulin analogs (i.e., insulin glargine and insulin detemirere) are produced to cover up the whole day insulin deficiency in patients with diabetes as opposed to the human regular insulin. Although both are different basal insulins, they bring great improvements in their mechanism of action and time of action as opposed to NPH insulin [41,42,43]. 

### 5.1. Insulin Glargine

**Figure 5:**
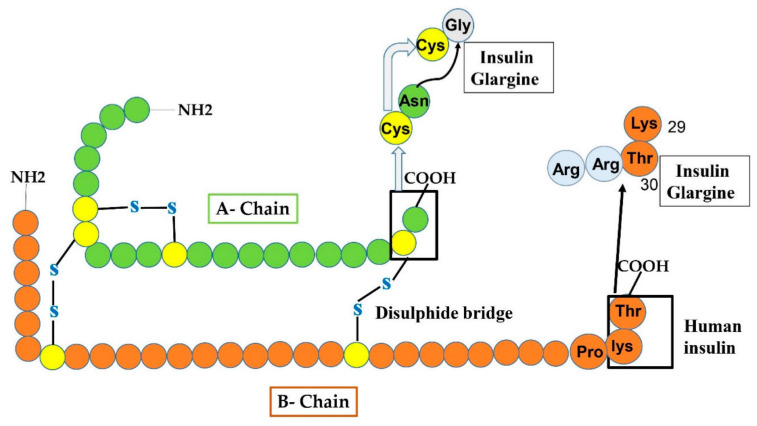
Manifests the production of insulin glargine from human insulin by amino-acids replacement at both A and B-chain. At A-chain, in the carboxy-terminal, Gly residue replaced the Asn residue at 21-site shown by an arrowhead. While at B-chain, two additional Arg amino acids are added.

This type of insulin was manufactured in 2000 and is an analog having a long-period of action of basal insulin administered in subjects having TIDM or TIIDM once a day. In Glargine, glycine replaced asparagine at position 21 of the A chain and two arginine amino acids are added at the C-end of the B chain (as shown in Figure 5). Such changes permit glargine to produce micro-crystal of hexameric form of insulin after its administration or injection. The addition of arginine amino acids alters the iso-electric point from PH 5.4 to 6.7 leading the substance to be water-soluble at acidic PH. A longer period of activity at neutral PH is nearly 24–26 h. Long-lasting activity of insulin glargine declines the peak effect reducing the possibility of occurrence of hypoglycemia [44]. During a shortage of natural insulin, it is necessary to inject glargine analog in fusion with an analog having long action taken with food to decrease post-prandial hypoglycemia. However, it cannot be fused with other sorts of insulin. It is not certified for use in children under the age of six. TIIDM is a major cause of cardiovascular disease, which may cause the death of TII diabetic subjects. The ORIGIN (outcome reduction with an initial Glargine intervention) investigation was devised and administered to estimate the level at which the glargine therapy could decline cardiovascular evidence in subjects with diabetes after targeting the blood glucose extent [45]. However, there is still a point of argument among scientists on how to clarify the outcomes of the ORIGIN struggles [46].

### 5.2. Insulin Glargine U300 (Gla-300)

Insulin glargine is another type of long-lasting insulin analog having the same anatomy, way of acting, and metabolism as Gla-100 [47]. Certain analysis has declared the same formulations of both the glargine, but the PK and PD evaluation of Gla-300 recommended a longer duration of glycemic control in patients with type-I diabetes in comparison to the Gla-100 (30 h vs. 29 h) [47,48]. When clinical trials were carried out at phase III, it has been reported that although Gla-300 has good safety results, high tolerability rate and similar glycemic control, on the other hand, a reduced rate of hypoglycemic incidences, specifically nocturnal incidences were seen [49]. When elderly patients having type-II diabetes were treated with the Gla-300, they have more glycemic control, minor evidence of hypoglycemia and lower weightage in contrast to Gla-100. Therefore for elderly patients having T1IDM, selection of Gla-300 would be a better solution [50,51]. 

### 5.3. Insulin Detemir

**Figure 6:**
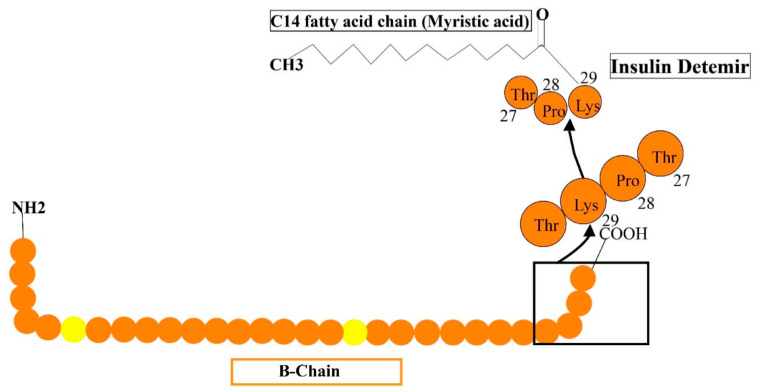
Reveals the formulations of detemir by the addition of some additional structures such as myristic acid is added to lys-29 to human insulin.

Long-acting (up to 24 h of activity) is a recombinant insulin analog, where the last residue (i.e., the threonine amino acid) is removed and fatty acid (meristic acid) is bonded covalently to the B29, permitting it to interact to albumin in the bloodstream [52]. It is manufactured by the expression of recombinant DNA in saccharomyces cerevisiae accompanied by some chemical alterations (Figure 6). The average timespan of action of detemir insulin is 5.7-h at the lowest dosage, 23.2-h at maximum dosage. Mostly, the advised amount of insulin detemir is once or twice/day. In clinical analysis, insulin detemir declined hemoglobin A1C (Hb AIC) to a specific extent of 7.0% for 70% of subjects such as human basal insulin NPH, and may cause a low rate of hypoglycemia in patients with TIDM and TIIDM [52]. 

### 5.4. Insulin Degludec

**Figure 7:**
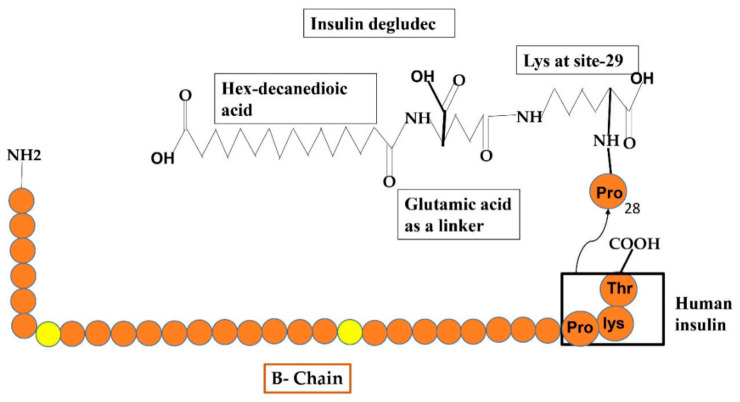
Reveals the formulations of Degludec insulin analog by the addition of some additional structures such as hex-decanedioic acid through Glu-linker is added to lys-29 of the B-chain of human insulin.

Another basal insulin analog that has an extra-long duration of activity with long-lasting glucose-reducing influence and lowered intra-subject instability following continuous 24 h basal insulin coverage by taking only a single dose. It has a different resorption phenomenon, which involves the production of multi-hexamers in under-skin tissues after taking a dose that additionally liberates various IDeg monomers (Figure 7). After PK/PD inquiry, it has been announced that IDeg has a lifespan of >25 h and renders to normal condition after three days of regimen [53]. This IDeg is also available in two forms just such as glargine i.e., 100 U/mL (U100) and 200 U/mL (U200). The random study under normal conditions with TIDM showed that both forms of IDeg were similar and glucose-reducing influences at the normal state were approximately between both compositions [54].

## 6. Fusion of Insulin Preparations (Two-Phase Insulins)

The notion of fusion of insulins was aroused to lessen the entire frequency of dosage taken per day during therapy with quick/intermediate insulin preparations for the satisfaction of the patients. Undoubtedly, the idea of thorough composition to imitate the natural human insulin encompasses a fusion of quick and basal insulin analogs but had not been feasible until now, as the basal insulin could not be fused with other insulins. Hence, for the preparation of two-phase insulin, a portion of quick-acting insulin has protamine to change an intermediate-acting insulin analog [55,56]. Two-phase human insulin contains several proportions of insulin with protamine equivalent. A checklist of frequently accessible premixed insulins involving: biphasic human insulin (BHI) in which 30% is regular human insulin along with 70% of protamine human regular insulin, BHI of aspart have 70% normal with 30% protamine human regular insulin designated as “BIAsp30”, BHI of lispro insulin that usually occurs in two forms, 25% combination of analog; 75% normal accompanied by 25% protamine, 50% fusion of analog; 50% of normal along with 50% of protamine [57].

Furthermore, other drugs such as dipeptidyl peptidase 4 (DPP-4) inhibitors having weight-neutral properties are being researched in teenagers. After providing an extra medication to an insulin-dependent patient’s regimen, glucose levels should be evaluated frequently, so that insulin dosing can be reduced if necessary. GLP-1 and other peptides are deactivated by DPP-4. As a result, inhibiting DPP-4 must enhance the level of intrinsic GLP-1, though they are unrelated to weight loss. DPP-4 inhibitors are oral drugs that have been used to treat adults with type-II diabetes mellitus since 2006 [58]. Alogliptin, linagliptin, saxagliptin, sitagliptin, and vildagliptin (not accessible in the United States) are some of the currently introduced DPP-4 inhibiting drugs. Recently, only a few studies using DPP-4 inhibitors in pediatric Diabetic subjects have been carried out. A comparison of type-II diabetes in children and adults revealed the pharmacokinetics and dynamics are comparable [59]. Within 12 weeks, the 1 mg dosage reduced HbA1c by 0.48%, whereas the 5 mg dosage reduced HbA1c by 0.63%. Further research is being conducted or is being planned. DPP-4 inhibitors are generally safe, with few adverse effects and little effects on weight. Issues about pancreatitis (pancreas inflammation) have emerged. Nevertheless, an analysis of the evidence has indicated that DPP-4 inhibitors do not cause pancreatitis [60].

Initially, the pharmacokinetics of insulin preparations had been determined indirectly based on the degree and duration of the hypoglycemia impact following injection. The approach for detecting the clearance of radiolabelled insulin from the subcutaneous incision site was established in the 1960s, and it was used to investigate the kinetics of insulin formulations [61]. This strategy presupposes that the pace of disappearance from the incision site equals the pace of appearance in the blood. For short-acting formulations, this assumption appears to be fulfilled with reasonable assessment, but it has been challenging to establish for long-acting formulations. After the usage of immunoassays for the measurement of insulin concentration, the present insulin formulations were assumed to be unable to replicate the 24-h profiles seen among normal persons [1]. The level of the fastest-acting soluble human insulin formulations rises relatively slow after administration, peaking after 2–3 h and remaining elevated for up to 6–8 h [62]. On the other hand, intermediate- and long-acting formulations based on amorphous or insulin crystal mixtures with or without protamine have too short and/or unpredictable profiles, which do not offer a consistent basal concentration between meals and at night. Present insulin formulations’ concentration profiles are governed by a considerably slower absorption protocol from the incision site to the blood and a quick dispersal and excretion following manifestation in the bloodstream. Thereby, the insulin concentration profile of the presently available human insulin analogs is identified by the absorption kinetics subcutaneously [62]. When insulin is injected subcutaneously, ~60% of insulin is degraded through the kidney, and ~30–40% is degraded through the liver [16].

## 7. Positive and Negative Impacts of Insulin and Its Analogs Usage against TIDM 

The introduction of insulin preparations (insulin analogs) has better protective effective outcomes and improved convenience of subjects than NPH insulin in subjects with type-I diabetes. Generally, the usage of insulin preparations having long-lasting action as basal insulin leads to rare incidences of hypoglycemia, as opposed to the NPH insulin usage [63]. An extensive survey of different analyses examining the earliest long-lasting insulin preparations in contrast with NPH insulin discovered a deduction in the possibility of nighttime hypoglycemia evidence and the hemoglobin A1c extent of subjects using the earlier [64]. An extensive alternative survey in subjects using ancient insulin preparations having long action compared to those using NPH insulins clarified such outcomes and presented low weightage [65].

### 7.1. Influences of Fast-Acting Insulins in Comparison to the Human Regular Insulin

Different efforts were made head-to-head which have revealed improved consequences through fast-acting insulin analogs as opposed to the regular human insulin. A comprehensive examination observed lesser variability among lispro, aspart, and human regular insulin for hemoglobin A1c and a lesser possibility of acute hypoglycemia with insulin preparations [66]. Certain findings discovered the equivalent frequency of wholly [67], nighttime, or acute nocturnal [68,69] hypoglycemic incidences through Lispro, or regular human insulin therapy. Nevertheless, alternatives discovered substantially declined nighttime hypoglycemic events through lispro compared to human regular insulin [70]. Other studies contrasting aspart insulin and human regular insulin (along with NPH insulin) observed a lesser but considerable reduction in HbA1c (hemoglobin A1c is a type of test in adults, that measures the average blood glucose level during the duration of last two to three months) and considerably lesser postprandial blood sugar extent through aspart at six [71,72], twelve [72,73], and thirty months [74]. Alternative analysis between aspart and human regular insulin along with basal NPH insulin obtained no considerable variations in HbA1c and acute hypoglycemic events during 12 or 64 weeks [75]. Administration of Glulisine along with the Glargine insulin fifteen minutes before the meal revealed considerably more decline in HbA1c as opposed to that by human regular insulin subjected 30–45 min before meals, while evidence of acute hypoglycemia was equivalent [76]. The efficiency of all fast-acting insulin preparations is entirely similar. Analysis at 26 weeks [77] compared Glulisine and Lispro insulins, no variability in the outcome was declared regarding declining in HbA1c and entire nocturnal hypoglycemic rate. However, in one study [78] with fast-acting insulin preparations used in CSII, slightly improved efficiency was observed with aspart insulin instead of the lispro and Glulisine. Anyhow, alternative analysis observed no substantial variation in aspart, Lispro, and Glulisine toward HbA1c, acute hypoglycemia, or any delivery system obstructions [79].

### 7.2. Influences of Long-Lasting Insulin Preparations

- Detemir insulin in comparison to NPH: The outcomes obtained after treatment with detemir or NPH insulins reveal the identical extent of HbA1c/FPS (fasting plasma sugar) [80,81,82,83,84], since detemir is correlated with the lesser possibility of hypoglycemia [81,83,84,85,86], and involving in night-time hypoglycemic evidence [80,81,82,84,85,86,87]. Analysis for two years observed somewhat lesser HbA1c extent through detemir therapy than NPH insulin, with additionally lesser FPG, primarily observed in an analysis of six months [85]. The weight reduction was usually seen after detemir therapy compared with the NPH insulin, an important feature of detemir insulin in individuals with type-I diabetes [80,82,83,84,85,86,87]. Some reports present administration of detemir for two times regularly more widespread than the subjection of once- regular detemir [88].
- Glargine-100 compared to Glargine-300: Glargine 300 shows similar glycemic control outcomes just like glargine-100, yet with rare nighttime hypoglycemic episodes. The Edition IV findings revealed comparable glycemic control through Gla-300 and Gla-100, but in the earlier eight weeks of therapy, the nighttime or acute hypoglycemic incidences were lesser and attained less weight with a variation of −0.6 kg through Gla-300 treatment [89]. However, after expanding six-months of Edition IV testing observed similar glucose control in both therapy categories and the same hypoglycemic evidence with Gla-100 and Gla-300 [90].
- Degludec insulin in comparison to glargine insulin: In many findings, the comparable decline in hemoglobin A1c [91,92,93,94,95] and gain of weight [93,94] was observed in subjects treated with Degludec in contrast to subjects who were treated with Gla-100. Anyhow, it was declared that treatment with Degludec showed identical [93,95] or lesser throughout hypoglycemic events and lesser or rare night-time hypoglycemic events [91,92,94,95] in comparison with the Gla-100.

However, after many analyses by clinicians, it has been proven that insulin Degludec is superior to insulin glargine in both diabetic diseases (i.e., diabetes type-I and II). Descriptive research in type-I diabetes has revealed insulin Degludec destined protective having declined hypoglycemic rate and equivalent glycemic control toward insulin glargine (analog) with long-lasting activity [96]. Trials by clinicians at stage III in Youngs with type-I diabetes [97] and type-II DM [98] manifest that control of glycemia was equivalent to insulin glargine at one year of investigation with lesser evidence of hypoglycemia. Since insulin Degludec has ultra- long-lasting action, it was analyzed using injections thrice a week compared to insulin glargine once a day and observed to have equivalent reaction [99]. The benefits of Degludec were discussed in various past publications [100,101,102]. To determine the difference between the efficiency and protective parameters of insulin Degludec and insulin glargine, both were injected regularly for one time, in basal-bolus treatment against type-I [97] and type-II [98] diabetes acclaimed useful glycemic control with lesser possibility of night-time hypoglycemia as opposed to insulin glargine. Therefore, approval of insulin Degludec by the FDA was carried out on the 26 September 2015 for glycemic control in children with the age of one year until adulthood [103].

d. Degludec insulin in comparison to the Detemir insulin: Certain face-to-face efforts have been made to compare the impacts of insulin degludec and detemir. Certain analysis declared that the outcome of both degludec and detemir in decreasing HbA1c was similar within 26 and 52 weeks [104,105,106]. In one analysis during this duration, the decline in FPG was substantially higher with degludec treatment [105] but not at 52 weeks in an alternative study [107]. Furthermore, it was declared that there was also substantially rare evidence of nighttime hypoglycemia per subject-year through degludec therapy in comparison with detemir. Yet, the comparable rate of entire accustomed episodes of hypoglycemia per subject-year during 26 and 52 weeks were obtained for both preparations [104,105,106,107].

### 7.3. Monitoring of Hemoglobin (Hb) A1c through Insulin Analogs

Control of hemoglobin A1c has become a standard marker to estimate an individual’s therapy success and conformity, as well as a standard framework for developing therapy-to-target objectives. By measuring HbA1c extent, indirect evaluation of entire glycemic subjection throughout the previous two to three months, with nearly 50% of the impact affected by the preceding 30 days, is possible [108,109]. Recently, two studies have demonstrated how fast-acting and long-lasting insulin preparations affect the extent of HbA1c [110,111]. One of these reviews having 49 randomized clinical analyses, compared an insulin preparation having fast action with regular human insulin, notable average variation of 0.1% in HbA1c was identified as endorsing preparations in subjects with type-I diabetes. No variation in HbA1c was observed between human insulin and fast-acting insulin analog among the subjects having type-II diabetes [111]. In the same literature with eight clinical analyses that compared the long-lasting activity of insulin preparation with NPH insulin, no significant variability in outcomes of HbA1c extent was obtained between these two insulin preparations [110]. Several analyses have evaluated the extent of HbA1c in subjects medicated with fused human insulins compared to those medicated with fused insulin analogs, involving biphasic insulin lispro constituents [112,113,114] and insulin aspart constituents [115,116,117]. Though one analysis revealed a less but substantial refinement in the extent of HbAc1 following therapy with 50/50 biphasic insulin lispro corresponding to biphasic human insulins, [114] other analyses did not succeed in obtaining identical benefits in favor of premixed insulin analogs [113,115,116].

Continually, nevertheless, subjects who were treated with biphasic insulin analogs showed improvements on PPG control reciprocal to biphasic human insulins, manifesting the elevated resorption frequency of the preparations [112,113,115,116,117].

### 7.4. Consequences of Insulin Preparations Regarding Special Considerations

#### 7.4.1. Children and Teenagers

Insulin treatment, especially basal-bolus treatment, is fundamental to the medication of teenagers and children having type-I diabetes just as with adults and is a pivotal molecule in the therapy of type-II diabetes. Though the influence of both the insulin glargine and insulin detemir has not been examined in infants, children, and teenagers having TII DM, both have been examined in infants and children with T1DM [118]. Both insulin glargine [119] and insulin detemir [120] have been revealed to be highly endured in children and youngsters with type-I diabetes and facilitate efficient control of glycemia accompanied by a remarkable decline in increasing blood glucose, yet not A1c extent, as opposed to NPH insulin. As agreed with the pharmacists, neither insulin glargine nor detemir can be fused with another insulin. Nevertheless, certain subjects (children), who desire to decrease inoculation numbers, may fuse their long-lasting basal insulin with their fast-acting basal (normal) insulin. Findings have been revealed that such a mixture of insulin preparations changes pharmacodynamic (PD) characteristics of the fast-acting insulin; the declined the apex effect and later duration of reaction [121,122].

#### 7.4.2. Pregnant Women

During pregnancy, most of the treatments without insulin are not recommended, and TIDM women need a suitable insulin administration with repeated changes during the whole period of pregnancy. Insulin is designated to be the most standard therapy for pregnant women having TIDM and diabetes during pregnancy whose hyperglycemic conditions sustain uncontrolled after alterations in food plan and lifestyle. While insulin detemir is designated in pregnancy, insulin glargine is specified only where prospective advantages surpass the threat due to the deficiency of potential randomized efforts in pregnant women. It has been shown that insulin detemir is superior to the NPH insulin regarding the accomplishment of A1C in afterward pregnancy in TID women, having lesser glucose levels in plasma and an equivalent rate of hypoglycemia [123] and bearable accompanied by NPH insulin having no determined protective outcomes [124]. It has been recommended that insulin glargine consumption during the pregnancy period should be appraised considering the advantages of enhanced control of glycemia compared to the NPH insulin [125]. Anyhow, researchers of presently published literature deduced that there is no incidence to endorse a single insulin therapy administration over the others during pregnancy [126]. It is necessary to keep in mind that such literature depends on the restricted number of clinical reports having fewer numerals of subjects being subjected to insulin glargine.

#### 7.4.3. Aged People

In joint studies of nine medicinal findings, insulin glargine was linked with improved glycemic control with a declined possibility of hypoglycemia in aged/elder subjects having type-II diabetes compared to others [127]. Insulin detemir has been revealed to be more efficient in people of 65 years of age though an elevated number of subjects observed hypoglycemic conditions at the initial stage [128].

## 8. Side Effects of Using Insulin Analogs (Negative Aspects)

It is no doubt the case that several studies have scrutinized the treatment influences of analogs of insulin about the onset of action and period of potency. However, certain analyses have demonstrated that some insulin analogs have hostile drug reactions. Short-acting insulin analogs were disclosed for their opposite effects involving hypoglycemia, impaired gait, tiredness, roseola, and bilateral leg edema [129]. However, insulin aspart and insulin lispro, which are the fast-acting insulin analogs, have revealed approximately 20% reduced possibility of hypoglycemic events in comparison to the regular insulin [130,131]. Insulin analogs have also been related to weight gain aided by their anabolic effects, mild or moderate edema, and cardiopulmonary congestion [132]. Nevertheless, constant advancement in insulin purification and preservation methods remarkably reduced the evidence of confined adverse impacts [133], even as some current outcomes from clinical trials showed that the use of insulin analogs has been observed to be secure, highly useful, and linked with remarkable refinement in subject comfort [134,135]. Still, continual advancement must abstain from the announced opposite effects of insulin preparations. The improved insulin analogs (i.e., the premixed insulin analogs) remarkably declined the possibilities of hypoglycemia, particularly nocturnal evidence, gaining weight, and other adverse effects [16,136].

## 9. Some Obstacles Faced by Patients with Diabetes through Insulin Therapy

Ultimately, everyone having diabetes type-I, and, importantly, having diabetes type-II, need insulin treatment. Previously insulin administration had been considered as “one sort and standard for all,” but not in this era, it has been recommended to have self-care-dependent strategies for better control of glycemic conditions [137,138]. Acute treatment of insulin that needs immediate management has certified long-lasting advantages, but the possibility of acute hypoglycemia may affect overall quality of life [139].

Although the most appropriate treatment method is insulin preparations accessible to monitor the hyperglycemic condition, subjects with diabetes have to face various difficulties involving some complications in administering insulin, inconvenience after injection, etc. [140,141]. Therefore, to avoid all these complexities, it becomes necessary to address progressed and effective techniques for insulin delivery. Advanced technologies for transferring insulin fulfill insulin delivery appropriately with reduced insensitivity. Such technologies have successfully influenced the subject’s awareness of insulin therapy in addition to a better life standard [142]. Primarily, large and massive needles were used for the delivery of insulin. Becton Dickinson had discovered the primarily specified syringe in 1924 [143]. After that, many advances in this syringe were made to improve the delivery system such as novo syringe [144], plastic syringes [145], U-100 plastic syringe [145], U-500 insulin syringe [146], etc. Despite all these advances in syringes, patients felt inconvenience in subjecting insulin multiple times in a day. [147]. Furthermore, the use of such syringes was linked with less precision of dosage, discomfort, and delivery challenges [146,148]. These negative impacts created many obstacles for patients to control glycemia [149]

## 10. The Introduction of Other Methods for Insulin Delivery to Fight against These Challenges

Many trials have been carried out to establish various ways of insulin delivery that are secure, effective, with no need for injections. Several other approaches for insulin delivery, besides inoculation of insulin, involve the administration of insulin tablets orally to resist digestion of insulin in the gastro-intestinal tract, inbreathe insulin, insulin pumps, and biosynthetic pancreases.

The administration of insulin tablets orally would transfer the dosage directly to the hepatic portal passage, imitating the naturally produced insulin hormone. Insulin is expected to be transferred to the liver directly, monitoring hepatic glucose formation, a crucial factor to hyperglycemia in type-II diabetics. Furthermore, oral insulin regimens have the probable advantages of elevating the subject’s agreement. Hence, several in vitro experiments have elaborated on how to transport adequate ideal insulin from the gut to the portal vein to cope with blood glucose levels [150].

In October 2012, a medical analysis including contributors examining the protective, suitable, PK, and PD features of an oral insulin preparation was fruitfully accomplished by Novo Nordisk in association with Danish producer Merrion Pharmaceuticals [151]. Other methods were also proposed for diabetes treatment. The purpose of both methods is to provoke basal insulin secretion. The first method is continuous subcutaneous insulin infusion (CSII), through which a defined dose of insulin is carried through the small pump to imitate the basal insulin production. The replacement of basal insulin through the CSII method is adaptable at about a 30 min gap according to the need of patients throughout the whole day. Another method is multiple daily injections (MDI) that facilitate the replacement of basal insulin through the combination of long-lasting insulin preparations along with separate subjection of fast-acting insulin analog at the time of the meal. Thus, such a method usually depends on >four daily injections [152]. Anyhow, the main ambition is to discover a synthetic pancreas having a capacity of 100% TIR (time in range), 0% period before the range, and inexpensive for all individuals. Though this assessment requires expansive adherence and period, it can modify insulin treatments [144].

## 11. Upcoming Directions Concerning Insulin Therapy

Investigations and advancement upon insulin analogs with improved features, such as an improved way of conveyance or activation are in progression. The administration of insulin orally through the portal vein prevents the subjects from the injection and may appropriately convey insulin by the resorption through the portal vein [152]. However, some findings have illustrated no considerable variability between orally and injected insulins about pharmacokinetic PK, PD, or protective measures. More efforts are still needed [153]. Moreover, despite the advancement in efficiency, protective measures, and different delivery methods, and even with the subject’s satisfaction (convenience) and adjustability, some demands are still present. Regarding the total health-remedy prices, insulin preparations and older insulin therapies are high-cost, since the therapy through insulin preparations is correlated with life excellence and expectation of life. Additionally, declined probability of hypoglycemia, higher satisfaction, and in certain cases, low weightage represent the advantages facilitated by insulin preparations as opposed to human insulin.

## 12. Conclusions

Incredible advancements in the field of biotechnology have proved/provided miraculous options for curing different chronic diseases, such as diabetes Mellitus disorder (which is prevailing very fast in the human population). Scientists and investigators had to face many challenges during the development of insulin therapy, and it took more than 100 years since the 19th century to improve insulin therapy against diabetes. Through modern technologies and interventions, and using recombinant DNA technology, scientists, and researchers could introduce insulin analogs for the treatment purpose against TIDM and TIIDM. After analyzing all of the findings regarding the consequences of insulin analogs, it became clear that recombinant insulin has a critical role in the treatment of diabetes. Additionally, a reduced possibility of hypoglycemia, greater satisfaction and in certain cases, and low weightage has been observed through insulin preparations. Although there were some adverse effects of insulin preparations and some obstacles were there for a patient with diabetes for insulin delivery, with advancements, scientists were able to reduce such negative impacts. Moreover, for the delivery of insulin, different new methods are established for patients’ convenience. Still, trials are in progress to further improve insulin treatment against diabetes (such as the introduction of commercial pancreases).
